# Deep Learning Based Automated Orthotopic Lung Tumor Segmentation in Whole-Body Mouse CT-Scans

**DOI:** 10.3390/cancers13184585

**Published:** 2021-09-13

**Authors:** Wouter R. P. H. van de Worp, Brent van der Heyden, Georgios Lappas, Ardy van Helvoort, Jan Theys, Annemie M. W. J. Schols, Frank Verhaegen, Ramon C. J. Langen

**Affiliations:** 1 Department of Respiratory Medicine, NUTRIM—School of Nutrition and Translational Research in Metabolism, Maastricht University Medical Center+, 6229 ER Maastricht, The Netherlands; ardy.vanhelvoort@danone.com (A.v.H.); a.schols@maastrichtuniversity.nl (A.M.W.J.S.); r.langen@maastrichtuniversity.nl (R.C.J.L.); 2 Department of Radiation Oncology (MAASTRO), GROW—School for Oncology and Developmental Biology, Maastricht University Medical Centre+, 6229 ER Maastricht, The Netherlands; brent.vanderheyden@maastro.nl (B.v.d.H.); georgios.lappas@maastro.nl (G.L.); frank.verhaegen@maastro.nl (F.V.); 3 Danone Nutricia Research, 3584 CT Utrecht, The Netherlands; 4 Department of Precision Medicine, GROW—School for Oncology and Developmental Biology, Maastricht University Medical Center+, 6229 ER Maastricht, The Netherlands; jan.theys@maastrichtuniversity.nl

**Keywords:** lung cancer, lung tumor segmentation, artificial intelligence, deep learning, µCBCT

## Abstract

**Simple Summary:**

The development of more translatable orthotopic mouse models is essential in order to study lung cancer more realistically. However, a major challenge in these orthotopic mouse models is the monitoring of tumor take, tumor growth and the detection of therapeutic effects. Therefore, the aim of this study was to train and validate a deep learning algorithm for fully automatic lung tumor quantification in whole-body mouse µCBCT scans. This deep learning application enables highly accurate longitudinal evaluation of tumor volume changes in mice with minimal operator involvement in the data analysis. In addition to longitudinal quantification of tumor development, the algorithm can also be deployed to optimize the randomization and 3R animal welfare aspects of the experimental design in preclinical studies.

**Abstract:**

Lung cancer is the leading cause of cancer related deaths worldwide. The development of orthotopic mouse models of lung cancer, which recapitulates the disease more realistically compared to the widely used subcutaneous tumor models, is expected to critically aid the development of novel therapies to battle lung cancer or related comorbidities such as cachexia. However, follow-up of tumor take, tumor growth and detection of therapeutic effects is difficult, time consuming and requires a vast number of animals in orthotopic models. Here, we describe a solution for the fully automatic segmentation and quantification of orthotopic lung tumor volume and mass in whole-body mouse computed tomography (CT) scans. The goal is to drastically enhance the efficiency of the research process by replacing time-consuming manual procedures with fast, automated ones. A deep learning algorithm was trained on 60 unique manually delineated lung tumors and evaluated by four-fold cross validation. Quantitative performance metrics demonstrated high accuracy and robustness of the deep learning algorithm for automated tumor volume analyses (mean dice similarity coefficient of 0.80), and superior processing time (69 times faster) compared to manual segmentation. Moreover, manual delineations of the tumor volume by three independent annotators was sensitive to bias in human interpretation while the algorithm was less vulnerable to bias. In addition, we showed that besides longitudinal quantification of tumor development, the deep learning algorithm can also be used in parallel with the previously published method for muscle mass quantification and to optimize the experimental design reducing the number of animals needed in preclinical studies. In conclusion, we implemented a method for fast and highly accurate tumor quantification with minimal operator involvement in data analysis. This deep learning algorithm provides a helpful tool for the noninvasive detection and analysis of tumor take, tumor growth and therapeutic effects in mouse orthotopic lung cancer models.

## 1. Introduction

Lung cancer is the leading cause of cancer deaths worldwide, comprising approximately 25% of all cancer deaths [1]. Muscle wasting as a component of cancer cachexia is frequently observed in lung cancer patients and is associated with poor clinical outcome and decreased overall survival [2,3,4,5]. Preclinical research is essential for the development and evaluation of novel therapies targeting the tumor and the host. One of the greatest challenges encountered by researchers in preclinical lung cancer (cachexia) research is the translatability of animal models [6,7]. In order to study lung cancer more realistically, models in which the tumor grows in its original stroma where it can interact with the complex microenvironment are needed [8]. Such orthotopic lung cancer models have been described in mice [9,10,11], but are not widely used in preclinical research due to a range of challenges involved. A major challenge is the monitoring of tumor take and growth in these orthotopic lung tumor models. Unlike subcutaneous xenograft models, the tumors in orthotopic models are not visible to the eye and accessible for caliper measurements. Instead, a proxy for tumor progression in orthotopic mouse models is mostly obtained from histological assessment in terminal experiments ending at sequential time points [12,13]. However, this approach introduces variation and the number of required animals increases proportionally with each additional time point, which is not consistent with the 3R principle embedded in national and international legislation and regulations on the protection of animals used for scientific purposes [14]. A more suitable option is the use of noninvasive imaging, such as computed tomography (CT), to monitor tumor progression over time.

Micro cone beam CT (μCBCT) imaging plays an increasingly important role in preclinical cancer research. Previously, this imaging modality has been used successfully to detect tumors in lung cancer mouse models [15,16,17,18,19,20]. Analysis of the acquired imaging data by delineating the region of interest (ROI), in this case the lung tumor, allows researchers to extract quantitative information, such as tumor shape and tumor size. Traditionally, tumor segmentation is performed manually by delineating the tumor outlines in each slice of the three-dimensional (3D) CT image. However, this is labor intensive and time consuming, especially in case of repetitive CT measurements for longitudinal follow-up of tumor growth. In addition, manual delineation is a challenging task, as it requires great knowledge in anatomy and CT imaging, and it is susceptible to inter- and intra-observer variabilities. Semi-automated methods (such as thresholding and region growing) for the segmentation of lung tumors have been reported in literature already [21,22,23]. As such, robust and time-efficient automatic image segmentation methods to quantify tumor development in preclinical orthotopic models will offer great benefits for the research field.

Multiple reference atlas-based approaches have been used for automatic segmentation of organs [24,25]. However, these automatic approaches often do not achieve adequate segmentation quality for heterogenic ROIs, which typically apply for tumors. The implementation of artificial intelligence (AI) in medical image processing suggests that convolutional neural networks, such as U-Nets, are an appropriate foundation for automatic ROI segmentation. Previously, we developed a deep learning algorithm trained to automatically segment the mouse calf muscle complex on μCBCT images, to derive muscle mass [26]. This algorithm showed a high correlation with the actual muscle wet mass measurements. More recently, Schoppe et al. open-sourced a deep neural network called AIMOS for automatic multi-organ segmentation of normal organs in whole-body scans of mice [27]. This model was more than two orders of magnitude faster than the atlas-based approaches, without compromising on quality. These recent developments suggest that AI will be a great platform to develop a fully automatic segmentation workflow for the quantification of lung tumors in CT images.

Here, we present the development and validation of a deep learning algorithm, based on existing AI architecture, for automated lung tumor segmentation in whole-body mouse µCBCT scans. This algorithm will be shown to be more consistent compared to human annotators and reduces the analytical workload to determine tumor size notably. In addition to longitudinal quantification of tumor development, our approach can also be deployed in parallel with the previously developed algorithm for muscle mass quantification, and to optimize the randomization and 3R animal welfare aspects of the experimental design in preclinical studies.

## 2. Materials and Methods

### 2.1. Mouse Experimental and Imaging Procedures

#### 2.1.1. Animals

Sixty male mice of 10 weeks (129S2/SvPasCrl, Charles River Laboratories) were socially housed (*n* = 3 in GM500 IVC cages (Tecniplast, Buguggiate, Italy) with corn cob bedding (JRS Lignocel, Rosenberg, Germany)) in a climate-controlled room (12:12 dark-light cycle with a constant room temperature of 21 ± 1 °C). Mice were given ad libitum access to food (AIN-93M, Bio-Services BV, Uden, NL, USA) and drinking water (autoclaved, softened and acidified (pH = 2.5)). After 1 week of acclimatization, animals underwent surgery at a standardized time window during their inactive period of the day. Following surgery, animals were monitored daily. Data shown were derived from the combination of several experimental cohorts with identical animal characteristics and experimental procedures. The CT images of in-total 60 mice were used to create training and validation datasets.

This work was conducted in accordance with institutional guidelines for the care and use of laboratory animals established by the Ethics Committee for Animal Experimentation of the Maastricht University, in full compliance to national legislation on animal research following the European Directive 2010/63/EU for the use of animals for scientific purposes and was part of a set of experiments to establish an orthotopic model of lung cancer cachexia.

#### 2.1.2. Tumor Model

Murine K-ras^G12D^; p53^R172HΔG^ lung epithelium-derived adenocarcinoma cells [28], kindly provided by Dr. J.M. Kurie, were cultured in vitro with Roswell Park Memorial Institute 1640 medium (Gibco, Rockville, MD, USA), supplemented with 9% fetal bovine serum (Gibco). Tumor cells were trypsinized in a sub-confluent state and suspended in growth factor reduced matrix (Matrigel, Corning Inc., New York, NY, USA) at a concentration 2 × 10^6^ cells/mL. Animals were anesthetized using a mixture of air and isoflurane (4% induction, 2% maintenance) and placed in a position of right lateral decubitus. Fur was removed and a 1 cm superficial skin incision was made below the left scapula. Fat was removed, and underlying muscles (excluding intercostal muscles) were carefully lifted. While visualizing the lung motion, 10 µL of tumor cells (2 × 10^4^ cells in 10 µL) were injected through the intercostal space into the lung. The muscles were placed back on top of the rib cage in the correct orientation and the skin was closed using a 5-0 suture. All mice receive pre-operative analgesia (Carprofen and Buprenorphine) via subcutaneous injection and post-operative analgesia (Carprofen) in the drinking water.

#### 2.1.3. Experimental Protocol

Body weight and food intake were measured daily at a standardized time window during their inactive period of the day. At baseline and weekly after surgery, µCBCT imaging was performed for all mice to assess lung tumor development and to detect muscle volume changes over time [26]. At the end of the experiment, when mice reached cachexia related humane endpoints, mice were scanned and subsequently sacrificed using intraperitoneal pentobarbital overdose. Lungs and skeletal muscles and other organs were collected using standardized dissection methods and frozen/fixed for further analysis.

#### 2.1.4. Animal Imaging

The anesthetized animals were placed in prone position with toes facing the flanks (foot and tibia angle ± 90°), and scanned using a µCBCT scanner (X-RAD 225Cx, Precision X-ray Inc., North Branford, CT, USA) at an X-ray tube potential of 50 kVp, X-ray tube current of 5.6 mA, and an imaging time of 2 min [29]. The imaging dose of 30 cGy was verified using a PTW TN300012 Farmer-type ionization chamber (PTW, Freiburg, Germany) according to the AAPM TG-61 protocol [30]. The µCBCT projection data was reconstructed using the Pilot Feldkamp back projection algorithm with a voxel dimension of 100 × 100 × 100 µm^3^ [31].

#### 2.1.5. Manual Segmentation

Sixty lung tumor segmentations in the left lung were annotated manually by a scientist with appropriate anatomical knowledge using the SmART-ATP software (Precision X-ray Inc. North Branford, CT, USA and SmART Scientific Solutions BV, Maastricht, NL, USA). The annotated CT data were split randomly in four batches of 15 mice CTs each to conduct a 4-fold cross-validation. To assess inter- and intra-observer variability, one batch of mice (*n* = 15) was manually delineated by two additional annotators. Detailed guidelines on the annotation of the tumor (See Supplementary Information) were employed to ensure reproducible annotations from all annotators.

### 2.2. Automatic Image Segmentation

#### 2.2.1. Deep Learning Algorithm

The automatic tumor segmentation model contains a preprocessing step, a deep learning algorithm and a post-processing step. The preprocessing step ensures that all µCBCT scans have a fixed input size. First, air voxels were cropped by thresholding (−800 Hounsfield Units [HU]) from the original reconstructed µCBCT volume [512 × 512 × *Z*]. The number of cranial–caudal slices in the µCBCT reconstruction is denoted as *Z*. Then, the well-defined appearance of the lungs in µCBCT images was used to automatically derive the center of the cropped image volume by means of simple thresholding (−600, −200 HU) to mask low-density regions. Additionally, morphological operations (erosion and dilation using a spherical kernel of 5 × 5 × 5) were applied successively to subtract undesired low-density regions from the binary lung mask (e.g., bowel gas or trachea), and to extract the three-dimensional position of the lungs. Second, the cropped µCBCT slices around the binary lung mask (256 × 256 × *Z*) were resized to a 128 × 128 × *Z*/2 CT volume with cubic interpolation to fit the graphics processing unit (GPU) memory. Subsequently, the resized and original µCBCT volumes were normalized between −400 and 1000 HU. Finally, to improve generalization of the network, data augmentation was performed on every µCBCT dataset and its corresponding segmentation, in every epoch. The data augmentation consisted of randomly sampled rotations (−15°, 15°) and shearing (−5, 5) operations.

An in-house developed supervised deep learning algorithm (two-step 3D U-Net) was trained to automatically segment lung tumors on µCBCT images of mice. The network architecture for both steps of the 3D U-Net was similar and consisted of three encoding layers to analyze the CT dataset, and three decoding layers to produce the tumor segmentation. Every encoding layer contained two 3 × 3 × 3 convolutions followed by a rectified linear unit. Subsequently, the spatial resolution was halved in every dimension by applying 2 × 2 × 2 max pooling. The decoding path was symmetrical to the encoding path, with the exception that max pooling was replaced by bilinear up sampling. The dropout ratio was set to 0.5, the number of epochs to 350, Adam was used as training optimizer [32], and the dice similarity coefficient (DSC) was adopted as optimization function to train our convolutional neural network. An illustration of the two-step 3D U-Net network architecture is shown in Figure S1.

The 3D U-Nets were applied successively in TensorFlow. The first step was applied on a rescaled low-resolution dataset (200 × 200 × 200 µm^3^) to define a tumor-specific cropped 3D volume-of-interest. In the second step, this position matrix was used on a cropped full-resolution dataset to segment individual foreground voxels. The two-step network was trained, tested, and validated on a NVIDIA Quadro P6000 (24 GB) GPU and made use of the NVIDIA CUDA^®^ Deep Neural Network library computational kernels.

The post-processing step consisted of a filling process, which was a unity check for holes in the automatic segmentation. No further post-processing steps such as filtering and morphological operations were performed.

#### 2.2.2. µCBCT to Mass Density Conversion

For the tumor mass calculation, a μCBCT to mass density conversion (CT2MD) was utilized to convert HU values from the reconstructed μCBCT scans to 3D density matrices (g/cm^3^). The CT2MD calibration curve was calculated based on scanning a cylindrical 30 mm diameter phantom dedicated for preclinical research in small animals (SmART Scientific Solutions BV, Maastricht, NL, USA). The phantom consists of a solid water structure enclosing 10 tissue-mimicking materials and 2 air holes with known and verified corresponding mass densities, e.g., ρ_solid-water_ = 1.02 g/cm^3^, ρ_adipose_ = 0.95 g/cm^3^, ρ_cortical bone_ = 1.33–1.82 g/cm^3^. In order to calculate the tumor mass, every voxel in the reconstructed μCBCT that belongs in the corresponding ROI on the binary output predicted by the automatic deep learning model was post-processed to obtain the mass density per voxel. Finally, the voxel dimensions, e.g., pixel spacing and slice thickness, derived by the μCBCT reconstruction settings were applied to determine the tumor mass.

## 3. Results

### 3.1. Quantitative Evaluation of the Segmentation Performance

**Figure 1:**
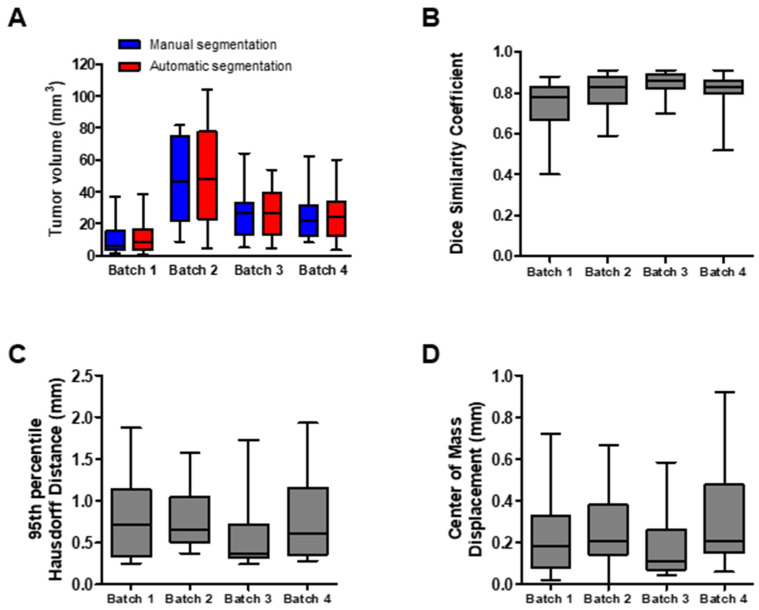
Quantitative performance metrics of deep learning algorithm. (**A**) Box plots showing the distribution of the tumor volumes in mm^3^ per cross-validation batch (*n* = 15), determined by manual and automatic segmentation. (**B**–**D**) Box plots of (**B**) the dice similarity coefficient, (**C**) 95th percentile Hausdorff Distance (in mm), and (**D**) the center of mass displacement (in mm), calculated for each of the batches.

In order to evaluate the performance of the automatic tumor segmentation algorithm, a four-fold cross-validation methodology was adopted. The lung tumor, ranging from 1.1 mm^3^ to 81.6 mm^3^, was manually delineated in 60 mice. The dataset was randomly partitioned into four equal sized batches (Batch 1–4) consisting of 15 mice each. One batch was retained as the test dataset to evaluate the model on unseen data, and the remaining three batches were used as training/test dataset. This cross-validation process was repeated four times, with each of the batches used once as the test dataset. High overlap in the tumor volumes was observed comparing the manual segmentations vs. the automatic segmentations (Batch 1: 10.19 ± 10.89 mm^3^ vs. 12.3 ± 11.69 mm^3^; Batch 2: 44.79 ± 26.28 mm^3^ vs. 46.76 ± 30.12 mm^3^; Batch 3: 27.14 ± 16.17 mm^3^ vs. 26.25 ± 15.53 mm^3^; and Batch 4: 26.25 ± 17.34 mm^3^ vs. 25.10 ± 15.72 mm^3^) (Figure 1A). Subsequently, three quantitative performance metrics (dice similarity coefficient (DSC), 95th percentile Hausdorff distance (95HD), and center of mass displacement (ΔCOM)) were calculated for each batch, which allowed quantitative evaluation of the performance of the automatic lung tumor segmentation algorithm (Figure 1B–D). The average DSC (±1 SD) of the four batches was equal to 0.80 ± 0.10, the average HD (±1 SD) was equal to 0.74 ± 0.48 mm and the average ΔCOM (±1 SD) was equal to 0.30 ± 0.46 mm.

Deep learning algorithms are generally known to be fast methods for micro-CT image segmentation. Although the training of the deep learning algorithm (two-step 3D U-Net) requires an initial time investment, the processing time to segment one 3D whole-body scan of a mouse is substantially shorter compared to manual segmentation. For the automatic tumor segmentation algorithm, the first step of the two-step 3D U-Net was trained in 7 h and 18 min., and the second step was trained in 8 h and 6 min., resulting in a total duration of 15 h and 24 min. The automatic volumetric segmentation was 69 times faster: analysis of the tumor of one whole-body µCBCT took the deep learning algorithm only 9 s, whereas the manual tumor segmentation time for one mouse was approximately 8 min.

### 3.2. Qualitative Evaluation of the Segmentation Performance

**Figure 2:**
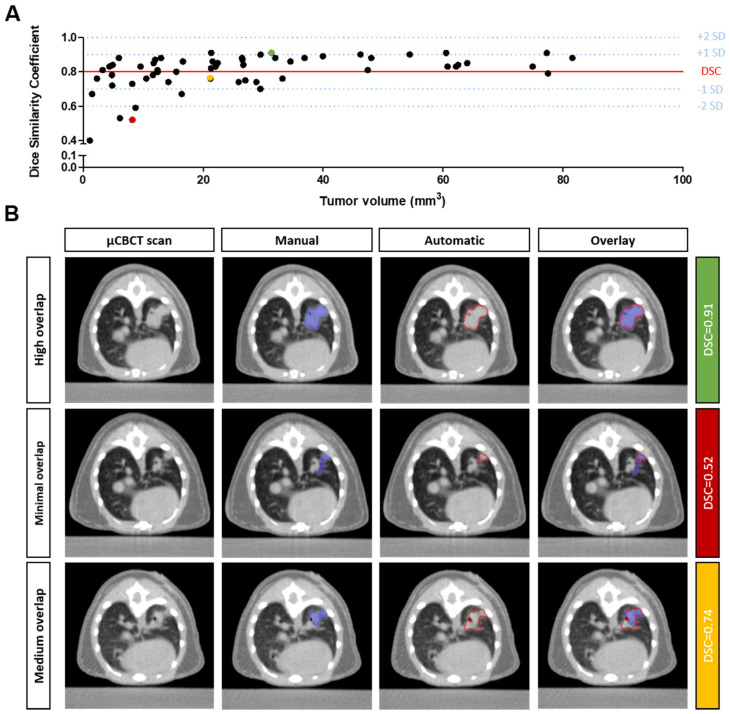
Qualitative evaluation of the algorithm performance. (**A**) Scatterplot of the DSC versus tumor volume (*n* = 60). The mean DSC is indicated with a horizontal red line. The ±1SD and ±2SD are indicated with blue dotted lines. (**B**) Axial µCBCT images showing (from left to the right) an unsegmented scan, the manual tumor segmentation, the automatic tumor segmentation, and the overlay of the manual and automatic segmentations. The first row shows an illustrative case with a high overlap, the second row shows a case with minimal overlap, and the third row depicts a case with medium overlap. The corresponding DSCs of each row in (**B**) are indicated in the scatterplot of (**A**) by the green, red and yellow markers.

Visual evaluation confirmed that the automatic tumor segmentations were in the correct anatomical location and closely overlapped with the manual delineations of the training dataset (Figure 2). However, as indicated by the quantitative performance metrics, in certain cases, there was a marginal deviation from the training dataset. To understand the origin of the deviation, we further examined automatic tumor segmentations in which the DSC was more than two standard deviations lower compared to the overall mean DSC (0.80; Figure 2A). Only 6.6% of the cases had a DSC less than 0.60. In all these cases, the tumor volume was less than 9 mm^3^. Several automatically segmented tumor volumes had a minor overlap with the manually delineated tumor volume, resulting in a low DSC (Figure 2B, row 2). In addition, in other cases, the automatic segmentation appeared correct, despite being different from the manually delineated training dataset (Figure 2B, row 3). This type of deviation is sometimes observed when the tumor is located in close proximity to the bronchi and/or pleura.

### 3.3. Assessment of Subjectivity and Bias in Human Interpretation

In order to assess the subjectivity and bias in tumor volumes based on human interpretation, we compared the manual delineations of the training dataset (annotator 1) with the manual delineations of a second and a third independent annotator on a subset of 15 µCBCT scans. In addition, we compared the segmented tumor volumes of all three annotators with the automatic segmentations of the deep learning algorithm (trained on manual delineations of annotator 1), using the Bland and Altman method. A significant correlation was observed comparing the manually segmented tumor volumes between annotators (Table 1, Figure S2A). However, a significant difference between the tumor volumes of annotator 1 vs. annotator 2 and annotator 1 vs. annotator 3 was observed, indicative of proportional bias or disagreement (Table 1, Figure S2B). This bias was absent when comparing the tumor volumes of annotator 2 vs. annotator 3 (Table 1, Figure S2B). Subsequently, three quantitative performance metrics (DSC, 95HD and ΔCOM) were calculated for each of the comparisons (Table 1, Figure S2C). The proportion of specific agreement (DSC) was similar comparing the segmented tumor volumes of all three annotators, respectively 0.76 ± 0.13 (annotator 1 vs. annotator 2), 0.76 ± 0.13 (annotator 1 vs. annotator 3) and 0.75 ± 0.13 (annotator 2 vs. annotator 3).

Comparing the manually segmented tumor volumes of all three annotators with the automatic segmentations of the deep leaning algorithm, consistently higher correlations were observed than between annotators (Table 1, Figure S2A). However, no agreement was observed between the tumor volumes of annotator 2 vs. deep learning and annotator 3 and deep learning algorithm (Table 1, Figure S2B). Importantly, good agreement between annotator 1 vs. the deep learning algorithm was achieved (which was trained on annotator 1′s contours). The DSC comparing the segmented tumor volumes of annotator 2 vs. deep learning and annotator 3 vs. deep learning were similar, respectively 0.74 ± 0.12 and 0.76 ± 0.10 (Table 1, Figure S2C). Although not significant, the DSC comparing annotator 1 vs. deep learning was higher (DSC = 0.81 ± 0.09).

With these analyses, we show that manual delineations of the tumor volume are sensitive to bias in human interpretation and that the algorithm learns according to the interpretations of the annotator of a training set.

### 3.4. Longitudinal Assessment of Tumor Volume

**Figure 3:**
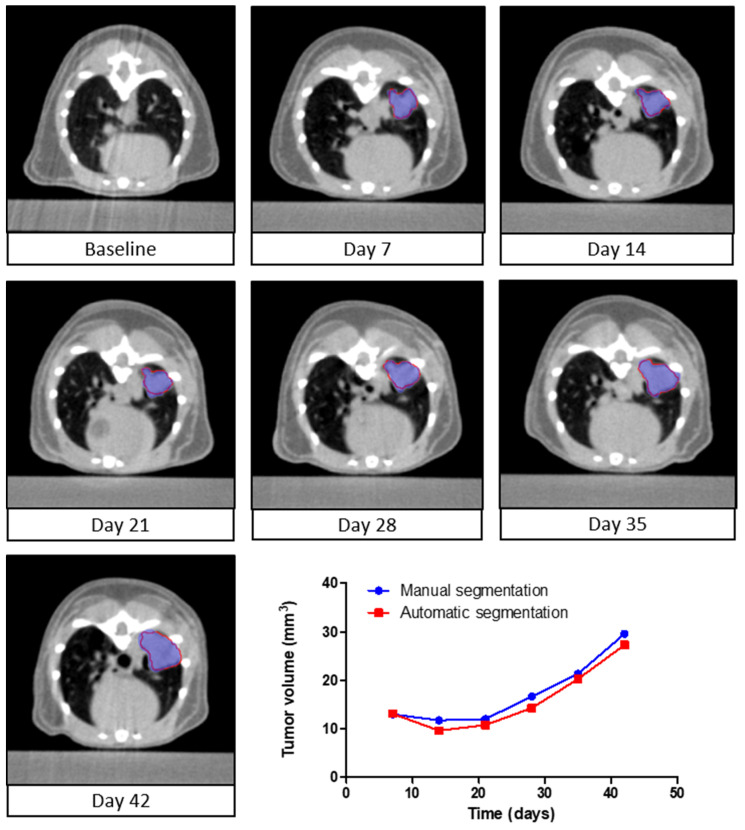
Longitudinal follow-up of tumor volume. A series of axial slices of µCBCT images showing the manual segmentation (blue) and automatic segmentation (red) of the lung tumor in a single animal versus time. The µCBCT images represent one mouse at seven different time points. The quantified manual (blue) and automatically (red) segmented volumes are presented over time in the line graph.

To study lung tumor development and treatments effects, longitudinal evaluation of tumor volume is essential. To evaluate automated follow-up capabilities of the deep learning algorithm, lung tumor volumes were assessed for serial µCBCT images of individual animals during the study. The volumes of the automatically segmented tumors were compared qualitatively and quantitatively to the volumes of the manually delineated tumors. Data of a representative series of µCBCT images is shown in Figure 3. The automatic segmentations closely overlap with the manual segmentations, and the average DSC (±1 SD) was equal to 0.88 ± 0.02. The total segmentation time of the deep learning algorithm was 1 min. compared to 1 h for the human annotator. These results show that the algorithm can be applied for the longitudinal follow-up of tumor development.

### 3.5. Simultaneous Quantification of Tumor Volume and Muscle Mass

**Figure 4:**
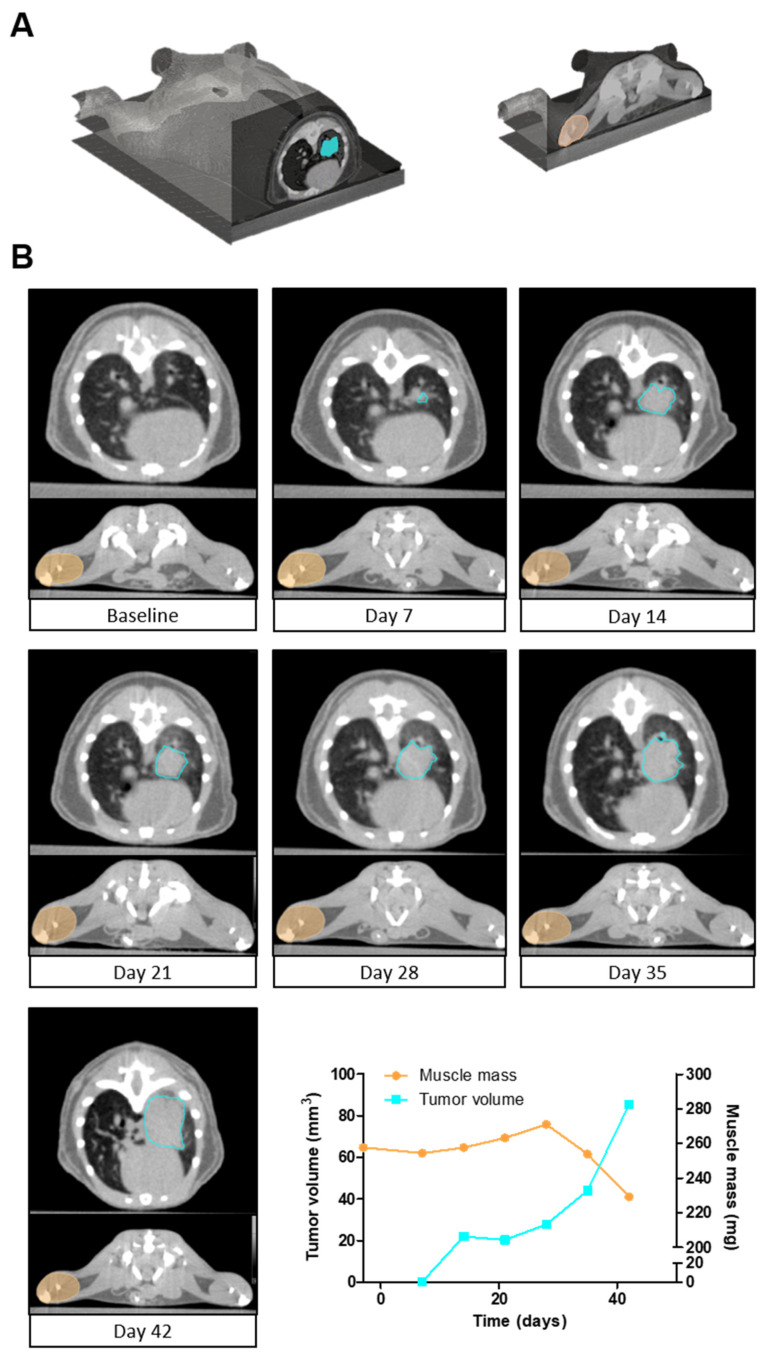
Longitudinal follow-up of tumor volume and muscle mass. (**A**) A 3D image of a whole-body mouse µCBCT scan indicating the region of the axial slices selected for (**B**). (**B**) A series of axial slices of µCBCT images illustrating the automatic lung tumor segmentations (light blue) and lower hind limb muscle complex segmentations (orange). The µCBCT images represent one mouse at seven different time points. The quantified tumor volumes (light blue) and muscle masses (orange) are presented over time in the line graph.

Muscle wasting as part of cachexia is often observed in lung cancer. Previously, we developed a highly accurate (R^2^ = 0.92, DSC = 0.93) deep learning algorithm for the quantification of hind limb muscle mass in mice [26]. Consequently, the muscle and lung tumor segmentation algorithm were executed sequentially to evaluate the feasibility of assessing both tumor and muscle mass longitudinally on serial µCBCT images (Figure 4). Experts visually verified the automatically segmented muscle and tumor volumes in the whole-body µCBCT scans. The automatic segmentation time was 13 s for both muscle and tumor. This equates to 1 min. and 31 s for an entire time series of µCBCT images of one mouse compared to 2 h and 47 min. of manual segmentation. Collectively, these data demonstrate the efficacy and feasibility of automated segmentation algorithms for the quantification of ROIs in preclinical research.

## 4. Discussion

Manual delineation of orthotopic lung tumors visualized by CT is not only a time-consuming task, but also requires knowledge of anatomy and CT-image analysis. Additionally, this task is susceptible to human error and bias. In this paper, we introduce a deep learning algorithm for automatic tumor volume segmentation in whole-body µCBCT images. With minimal reliance on operator involvement, our deep learning method performs fast and accurate volume-mass quantifications of orthotopically growing lung tumors in mouse models.

For the development of the automatic lung tumor segmentation algorithm, we applied and optimized a two-step 3D U-Net architecture. Since its introduction in 2015 by Ronneberger et al. [33], the 2D U-Net architecture is one of the most commonly used methods in biomedical image segmentation. Shortly after the introduction of the 2D U-Net, the network architecture was modified by Milletari et al. to a 3D U-Net architecture (or V-Net) for biomedical images that have a spatial relationship in all three dimensions [34]. Medical CT, CBCT, or MRI images are a great example of imaging modalities that benefit from a 3D architecture. Previously, the 3D U-Net architecture has been shown to be a proper method for organ [35] and tumor [36,37] segmentation in medical images. However, due to the increased number of 3D convolutions performed on a large medical imaging dataset, the GPUs available on the market today are still too limited in terms of memory. Therefore, we adopted a two-step 3D U-Net architecture. The first step was applied on a downsized dataset to find the approximate tumor location, and in the second step, this position matrix was used on a cropped normal sized dataset to segment the tumor on the highest resolution.

The newly trained deep learning algorithm for automatic tumor segmentation performs 69 times faster compared to manual segmentation, saving major amounts of time in operator involvement and data analysis. Following development of the model, the algorithm’s performance was assessed using specific quantitative metrics (e.g., DSC, 95HD and ∆COM) for comparison with the manual segmentations of the training dataset. The deep learning algorithm achieves good average performance scores (DSC = 0.80, HD = 0.74 mm and ∆COM = 0.24 mm). The performance scores of the lung tumor segmentation algorithm are slightly lower compared to the performance scores of the previously published muscle segmentation algorithm and AIMOS for normal tissue auto-segmentation [26,27]. This is not surprising, as there is wide variation in the size, shape and location of lung tumors in contrast to the muscle and other normal organs. Clearly, the low performance scores (mean DSC−2 SD) are restricted to tumors with a volume smaller than 9 mm^3^, particularly in small tumors located in close proximity to the bronchi or the pleura. In these scenarios, the acquired imaging data may contain insufficient information to distinguish between tissues, resulting in variation in the consistency of interpretation between the training dataset and the deep learning algorithm. Therefore, we recommend that for small tumors a human operator checks the results.

In accordance with previous studies [27,38,39], we found that manual annotations are sensitive to human bias in interpretation. Despite the use of detailed annotation guidelines, we showed that annotators recurrently disagree in the manual segmentations of the tumor volume. When the tumor is located in the middle of the lung free from other tissues, high agreement in the interpretation is observed. The variation in the interpretation is mainly caused by tumors located in close proximity to the bronchi. Where one annotator includes the bronchi as part of tumors the other exclude it from the delineation. Based on a relatively small dataset of only 60 manual segmentations, the agreement between the deep learning algorithm and the training dataset (annotator 1) is greater compared to human annotators. This may be due to the more consistent way in which the algorithm handles uncertainties compared to human annotators. These findings highlight the value of implementing AI in experimental preclinical and medical image segmentation. The bias in interpretation may also have an impact on the quantitative performance metrics of the algorithm if only manual segmentations of one annotator are used to create the trainings dataset. Although it is common practice in preclinical research to create training datasets generated by only one annotator [24,26,40,41], a training dataset based on two or more annotators will further increase the robustness of the model. Although it may not be necessary for the quantification of tumor volumes, this may be more important if the automatic tumor segmentations model is used for image guided high precision tumor irradiation [42,43]. Conversely, for irradiation, substantial geometrical margins are used to avoid under-irradiation.

This deep learning algorithm for lung tumor segmentation provides researchers with a helpful tool that enables fast, unbiased and interpretable quantifications of CT images. Several orthotopic mouse models have been described to study lung cancer [9,10,11]. What emerges unambiguously in these studies is that a tumor take of 100% is never achieved. In addition, without in vivo CT imaging, it is impossible to verify whether the tumor development is localized in the appropriate anatomical location, i.e., the lung parenchyma in our model, or if there is pleural seeding leading to the development of intra-pleural tumor growth. The automatic tumor segmentation algorithm overcomes these limitations and offers great benefits for the quality and consistency of the research. First, it detects if a tumor grows in lung parenchyma. If the algorithm does not detect a tumor (volume = 0 mm^3^), and this is confirmed by visual evaluation, mice can be excluded from an experiment reducing unnecessary distress for these animals. Then, the algorithm measures the tumor volume accurately, and subsequently, the mass of the lung tumor within one week following tumor inoculation. The tumor volumes can, for example, be used to randomize the animals into treatment groups prior to initiation of interventions, reducing variation and eventually the number of animals. In addition, we showed that the algorithm is capable of detecting changes in tumor mass over time. This is instrumental for the algorithm to be deployed as a valuable tool for preclinical lung cancer (treatment) research.

Finally, we showed that the automatic tumor segmentation algorithm can be used in combination with our previously developed algorithm for automated muscle mass determination [26]. Cancer cachexia, or muscle wasting, is a serious comorbidity and frequently occurs at advanced stage lung cancer [2]. Longitudinal assessment of both muscle mass and tumor volume will greatly aid in better understanding of the underlying mechanisms and interplay between tumor growth and muscle loss and allows studying the efficacy of therapeutic interventions on both phenomena in time.

## 5. Limitations

Despite the qualitatively high performance of the fully automatic AI-based lung tumor segmentation tool described here, limitations apply. Due to limited availability of µCBCT images, only 60 cases, the used dataset is small for deep learning applications. The deep learning algorithm is trained on the manual annotations of only one annotator. Furthermore, in this study, the deep learning algorithm was trained on a µCBCT image dataset collected at a single center and was acquired with one specific imaging protocol at an X-ray tube potential of 50 kVp, which can cause bias. Prior to implementation of the algorithm in other settings, further research is required to investigate whether the algorithm is valid on imaging datasets that were acquired at different X-ray tube potential settings, or on imaging datasets that were acquired with different spectral filtrations [31]. Finally, our training dataset was entirely based on 129S2/SvPasCrl mice. Nevertheless, we anticipate that the proposed algorithm is capable of segmenting tumor volumes in a variety of animals, as long as the study set-up (i.e., X-ray acquisition protocols and reconstruction settings) and animal characteristics are consistent. If the differences in the imaging parameters are too large, transfer learning can be applied, which will reuse the knowledge from the current model to facilitate the process of retraining the model on new datasets [44].

## 6. Conclusions

A deep learning algorithm was developed and validated to automatically segment orthotopic lung tumors in mice. It provides fast and highly accurate tumor segmentations with minimal operator involvement in data analysis. This deep learning algorithm is a helpful tool for the non-invasive detection and analysis of tumor take, tumor growth and therapeutic effects, and can be deployed to optimize the randomization and 3R animal welfare aspects of the experimental design.

## Figures and Tables

**Table 1:** Subjectivity and bias in human interpretation.

	Segmented Tumor Volume		Quantitative Performance Metrics		
	Correlation (R^2^) *	Agreement **	DSC	95HD (mm)	ΔCOM (mm)
Annotator 1 vs. Annotator 2	0.79 (*p* < 0.001)	No (*p* = 0.005)	0.76 ± 0.13	0.84 ± 0.63	0.51 ± 0.41
Annotator 1 vs. Annotator 3	0.88 (*p* < 0.001)	No (*p* = 0.011)	0.76 ± 0.13	0.82 ± 0.55	0.41 ± 0.54
Annotator 2 vs. Annotator 3	0.77 (*p* < 0.001)	Yes (*p* = 0.251)	0.75 ± 0.13	0.66 ± 0.47	0.39 ± 0.36
Annotator 1 vs. Deep learning	0.93 (*p* < 0.001)	Yes (*p* = 0.365)	0.81 ± 0.09	0.78 ± 0.53	0.30 ± 0.23
Annotator 2 vs. Deep learning	0.87 (*p* < 0.001)	No (*p* = 0.001)	0.74 ± 0.12	0.82 ± 0.41	0.51 ± 0.37
Annotator 3 vs. Deep learning	0.92 (*p* < 0.001)	No (*p* = 0.007)	0.76 ± 0.10	0.77 ± 0.39	0.45 ± 0.45

DSC: dice similarity coefficient, 95HD: 95th percentile Hausdorff distance, ΔCOM: center of mass displacement. * Linear regression. ** Agreement: One sample *t* test on the difference between annotators. Quantitative performance metrics are presented as mean ± SD.

## Data Availability

The code is licensed to a company (SmART Scientific Solutions BV). The company will further improve the product for commercial availability. The data presented in this study are openly available in zenodo at 10.5281/zenodo.5502007.

## Supplementary Materials

The following are available online at https://www.mdpi.com/article/10.3390/cancers13184585/s1, supplementary information: Manual annotation instructions. Figure S1: The two-step 3D U-Net network architecture implemented in TensorFlow. Figure S2: Subjectivity and bias in human interpretation.
