# Supplementary material for: Deep Learning Based Automated Orthotopic Lung Tumor Segmentation in Whole-Body Mouse CT-Scans
Source: Cancers (Basel). 2021 Sep 13;13(18):4585. doi: 10.3390/cancers13184585 (PMC8471805; doi:10.3390/cancers13184585)
Supplement: Supplementary file 1 [file cancers-13-04585-s001.zip › Supplementary figures_resubmission.pdf]

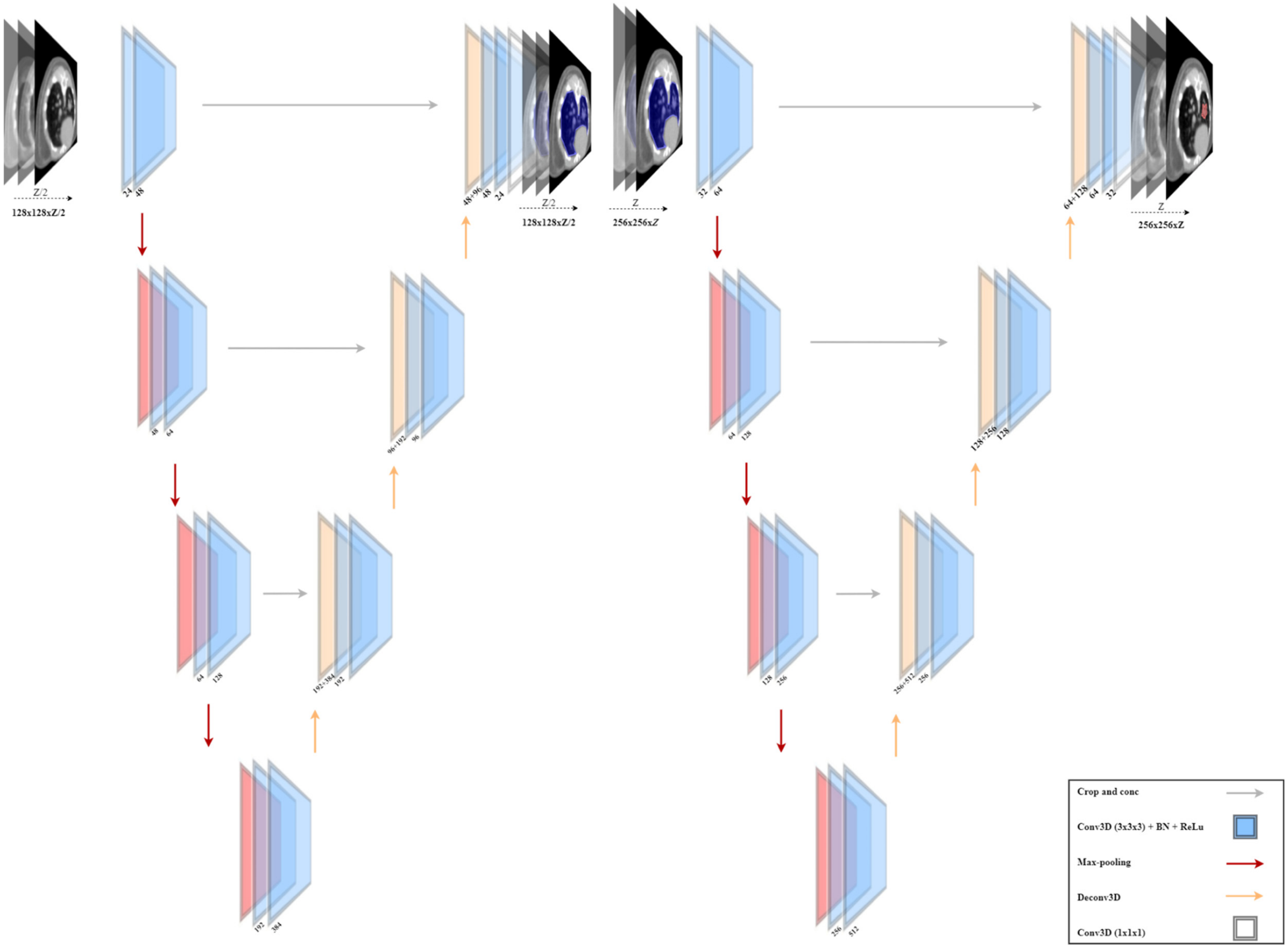

Supplementary Figure 1. *The two-step 3D U-Net network architecture implemented in TensorFlow.*

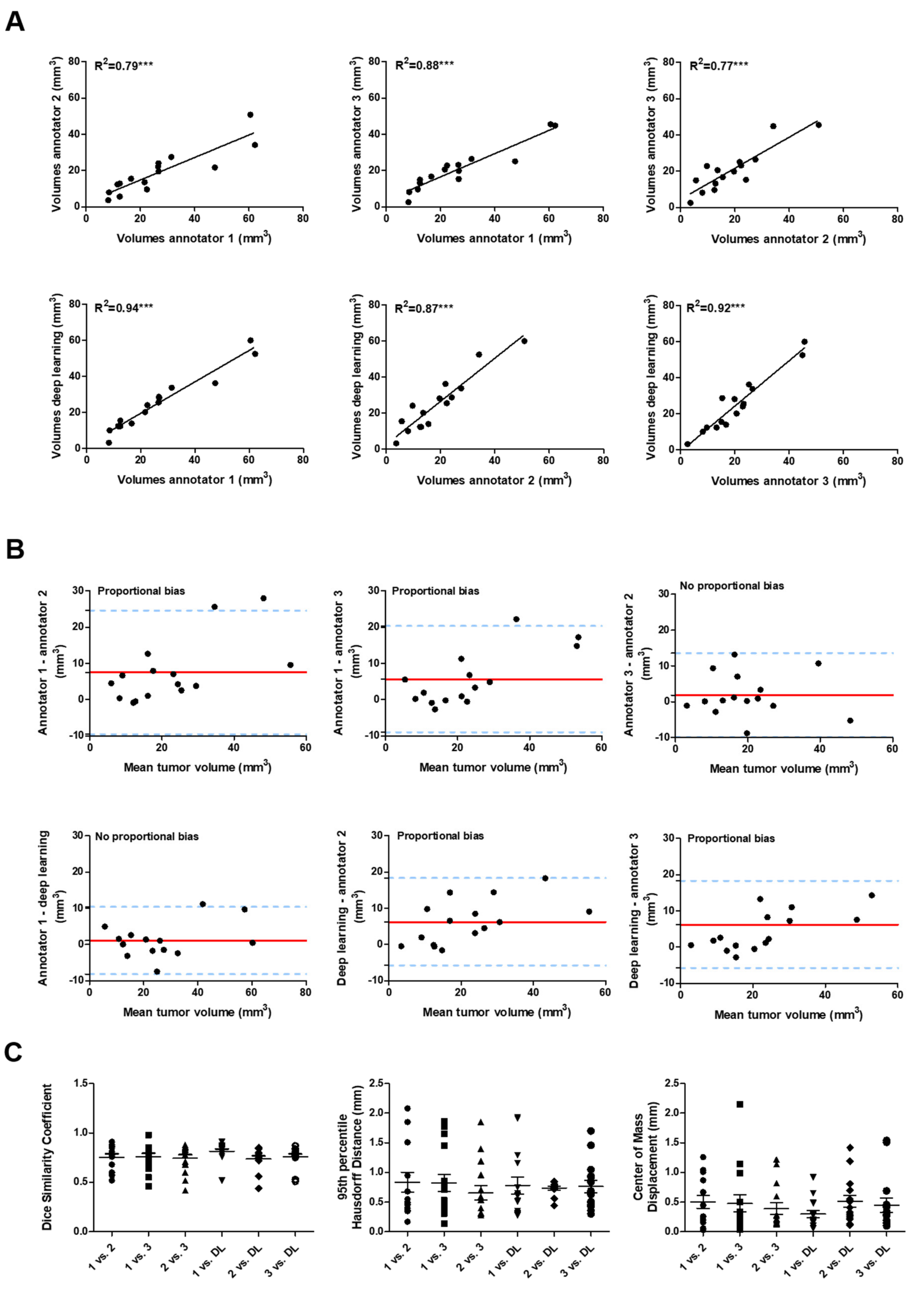

***Supplementary Figure 2. Subjectivity and bias in human interpretation. A**, Linear relationship between annotators and annotators-deep learning algorithm. *** P-value < 0.001. **B**, The Bland-Altman plot indicating agreement or disagreement between tumor volume segmentations. The dotted blue lines indicate the upper- and lower 95% confidence intervals. The red line indicates the mean. **C**, Aligned dot plot (n=15) of the quantitative performance scores*

(dice similarity coefficients, 95th percentile Hausdorff Distance (in mm), and center of mass displacement (in mm)) calculated for each of the comparisons.
