# Supplementary material for: Deep Learning Based Automated Orthotopic Lung Tumor Segmentation in Whole-Body Mouse CT-Scans
Source: Cancers (Basel). 2021 Sep 13;13(18):4585. doi: 10.3390/cancers13184585 (PMC8471805; doi:10.3390/cancers13184585)
Supplement: Supplementary file 1 [file cancers-13-04585-s001.zip › Supplementary information_resubmission.pdf]

Manual annotation instructions:

- **NOTE:** All manual segmentations should be performed on axial slices of the whole-body µCBCT.
- **Define the outer extensions of the lungs (figure 1).**
  - Lung volume ([-600, -200] Hounsfield Units (HU))
  - The ribs ([1000, 2000] HU) and the diaphragm ([250, 650] HU) form the outer layers of the lungs.

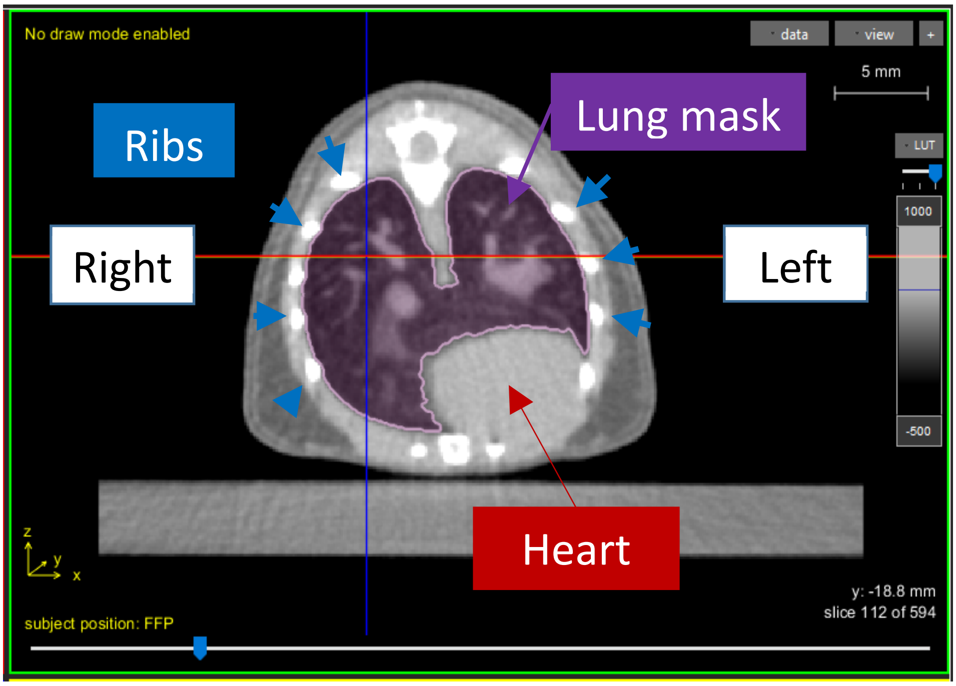

- **Define the outer extensions of the tumor <u>within</u> the left lung lobe (figure 2).**
  - Search for the tumor in the left lung lobe ([300, 600] HU; green selection).
  - Scroll to the middle of the tumor volume and manually delineate the structure (Drawing tools: Free hand or if possible 2D region growing).
  - Scroll through the tumor volume and determine the outer extensions cranial and caudal of the middle of the tumor volume. Use the coronal and median panel to determine the outer extensions (see green ruler)

- **Manually delineate the lung tumor volume**
  - Starting from the middle manually delineate all slices between the middle and outer extensions. If possible, use 2D region growing with a predefined threshold (300 HU)

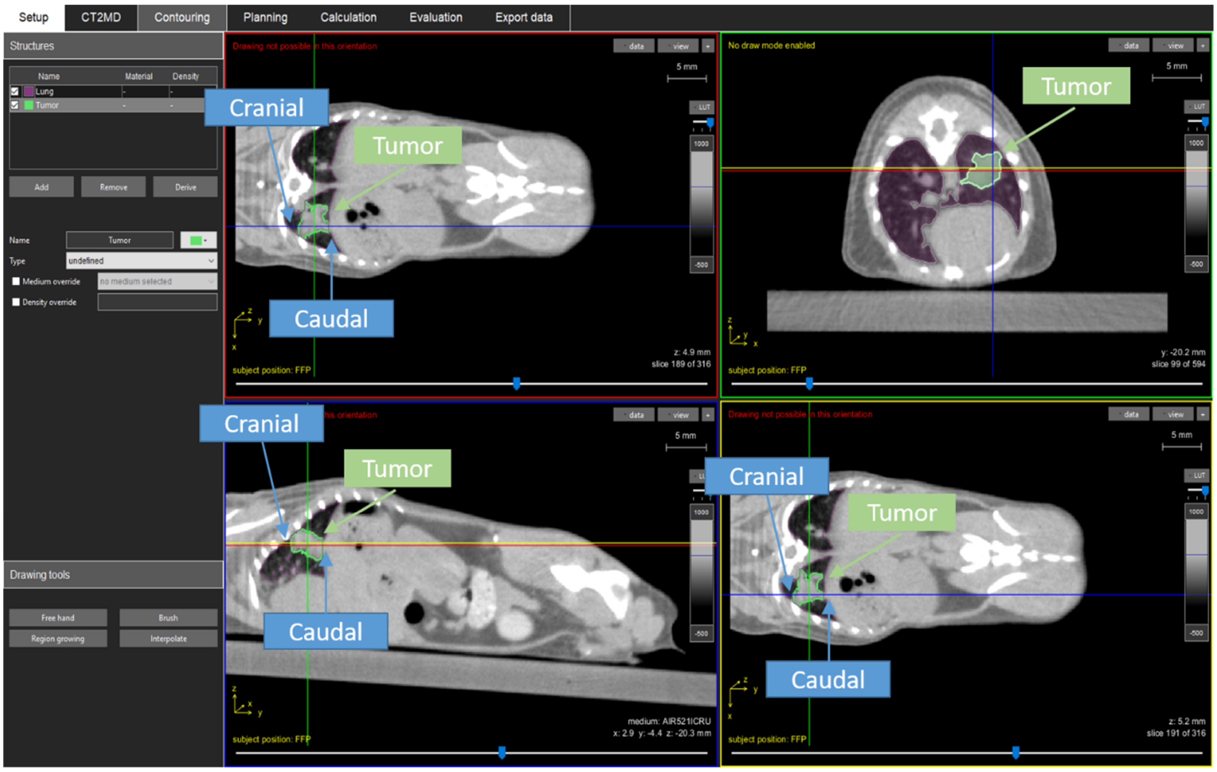
Setup
CT2MD
Contouring
Planning
Calculation
Evaluation
Export data
Structures
Name
Material
Density
Lung
Tumor
Add
Remove
Derive
Name
Tumor
Type
undefined
Medium override
no medium selected
Density override
Drawing tools
Free hand
Brush
Region growing
Interpolate
Drawing not possible in this orientation
No draw mode enabled
Cranial
Tumor
Caudal
Tumor
Cranial
Tumor
Caudal
Cranial
Tumor
Caudal
subject position: FFP
